# Differences Between Cancer-Specific Survival of Patients With Anaplastic and Primary Squamous Cell Thyroid Carcinoma and Factors Influencing Prognosis: A SEER Database Analysis

**DOI:** 10.3389/fendo.2022.830760

**Published:** 2022-03-10

**Authors:** Shuai Jin, Xiangmei Liu, Dandan Peng, Dahuan Li, Yuan-Nong Ye

**Affiliations:** ^1^ Bioinformatics and Biomedical Big Data Mining Laboratory, Department of Medical Informatics, School of Big Health, Guizhou Medical University, Guiyang, China; ^2^ School of Clinical Medicine, Guizhou Medical University, Guiyang, China; ^3^ Cells and Antibody Engineering Research Center of Guizhou Province, Key Laboratory of Biology and Medical Engineering, School of Biology and Engineering, Guizhou Medical University, Guiyang, China; ^4^ Key Laboratory of Environmental Pollution Monitoring and Disease Control, Ministry of Education, Guizhou Medical University, Guiyang, China

**Keywords:** ATC, PSCCTh, SEER database, prognosis factors, cox model, cancer-specific survival

## Abstract

**Purpose:**

Anaplastic thyroid carcinoma (ATC) and primary squamous cell carcinoma of the thyroid (PSCCTh) have similar histological findings and are currently treated using the same approaches; however, the characteristics and prognosis of these cancers are poorly researched. The objective of this study was to determine the differences in characteristics between ATC and PSCCTh and establish prognostic models.

**Patients and Methods:**

All variables of patients with ATC and PSCCTh, diagnosed from 2004–2015, were retrieved from the Surveillance, Epidemiology, and End Results Program (SEER) database. Percentage differences for categorical data were compared using the Chi-square test. Kaplan-Meier curves, log-rank test, and Cox-regression for survival analysis, and C-index value was used to evaluate the performance of the prognostic models.

**Results:**

After application of the inclusion and exclusion criteria, a total of 1164 ATC and 124 PSCCTh patients, diagnosed from 2004 to 2015, were included in the study. There were no differences in sex, ethnicity, age, marital status, or percentage of proximal metastases between the two cancers; however, radiotherapy, chemotherapy, incidence of surgical treatment, and presence of multiple primary tumors were higher in patients with ATC than those with PSCCTh. Further cancer-specific survival (CSS) of patients with PSCCTh was better than that of patients with ATC. Prognostic factors were not identical for the two cancers. Multivariate Cox model analysis indicated that age, sex, radiotherapy, chemotherapy, surgery, multiple primary tumors, marital status, and distant metastasis status are independent prognostic factors for CSS in patients with ATC, while for patients with PSCCTh, the corresponding factors are age, radiotherapy, multiple primary tumors, and surgery. The C-index values of the two models were both > 0.8, indicating that the models exhibited good discriminative ability.

**Conclusion:**

Prognostic factors influencing CSS were not identical in patients with ATC and PSCCTh. These findings indicate that different clinical treatment and management plans are required for patients with these two types of thyroid cancer.

## Introduction

Anaplastic thyroid cancer (ATC) is the most aggressive type of primary thyroid cancer, and accounts for approximately 1%–2% of all thyroid tumors, most of which occur in elderly adults. Giant cells, spindle cells, and squamous cells show obvious pleomorphism, while anaplastic tumors have no clear differentiation (1, 2). Primary squamous cell carcinoma of the thyroid (PSCCTh) is an extremely rare and highly aggressive malignant tumor, which comprises < 1% of all primary thyroid cancers and has poor prognosis. In terms of cytology, PSCCTh consists of large pleiomorphic epithelial cells, with frequent keratinization and necrotic components (3, 4). The most common symptom of PSCCTh is an enlarged anterior neck mass, followed by difficulty breathing or swallowing, and a change in voice. Both ATC and PSCCTh present with abundant squamous cell-like cells; hence, the two conditions are easy to confuse during clinical diagnosis and are treated similarly (5).

The similarities and differences between ATC and PSCCTh have become a focus of increased research attention. Given the rarity of these two diseases, conducting prospective studies with large samples is very difficult. Both types of primary thyroid cancer are highly aggressive and have poor prognosis. At present, combined treatments, including surgery, radiotherapy, and chemotherapy, are often used in the clinic (6); however, the role of adjuvant therapy for these two cancers remains unclear. In addition, differences in cancer-specific survival (CSS) between patients with ATC and PSCCTh have not been extensively studied, and understanding the factors that influence CSS prognosis in ATC and PSCCTh is crucial.

With the aim of better understanding the demographic and clinical treatment-related indicators that differentiate ATC and PSCCTh, and exploring factors influencing CSS and prognosis in patients with these tumors, we conducted a retrospective study based on data from the Surveillance, Epidemiology and End Results Program (SEER) database (SEER 18 registry grouping) (7).

## Materials and Methods

### Data Source

We extracted patient data from the updated SEER database (https://seer.cancer.gov), and downloaded using SEER*Stat software (version 8.3.6; https://seer.cancer.gov/), this database (Incidence -SEER 18 Regs Custom Data with additional treatment fields, Nov 2018 Sub, 1975-2016 varying) includes radiotherapy and chemotherapy information. Since patient identifying information has been removed from this data set, local ethics committee review and patient informed consent were not required.

### Study Population

The cases included in this study were diagnosed with ATC or PSCCTh from 2004–2015. Samples were selected based International Classification of Diseases for Oncology third edition (ICD-O-3) organizational behavior codes, issued by the World Health Organization (WHO) in 2013. The histology codes for ATC are 8012, 8020, 8021, and 8030–8032, and those for PSCCTh are 8070–8076; the primary thyroid location code for all samples was C73.9. Demographic and clinical data, including year of diagnosis, age at diagnosis, ethnicity, sex, primary tumor, marital status at diagnosis, surgery of the primary site, N stage, M stage, radiotherapy, chemotherapy, survival time, and SEER cause-specific death classification-related variables, were retrieved for analysis. Samples with unknown ethnicity or survival time, or where the surgical status or N stage data field was blank, were excluded from the analyses.

### Statistical Analysis

Categorical data are expressed as rates or percentages and differences were analyzed using the chi-square test. In this study, CSS of patients with ATC and PSCCTh was the primary research endpoint. The Kaplan-Meier method was used to evaluate patient CSS, and the log-rank method was used to assess differences in CSS between patients with the two types of thyroid cancer. Single-factor and multi-factor Cox regression models were used to assess the impact of demographic and clinical factors on CSS in patients with ATC and PSCCTh; variables with p < 0.2 on univariate test and important variables, recognized by clinical experts (such as treatments), were included in the multivariate model. Harrel’s Consistency Index (C-Index) was used to evaluate the models. Mapping and statistical analyses were conducted using R Language 3.6.0 (http://www.r-project.org/). All p-values were two-sided and p < 0.05 was considered significant.

## Results

### Differences in Demographic and Clinical Indicators Between Patients With ATC and PSCCTh

Of data in the SEER database from 2004 to 2015, 1288 patients with primary thyroid cancer (ATC = 1164, PSCCTh = 124) met the inclusion and exclusion criteria and were finally included in this study. Analysis of differences in demographic and clinical data of patients with ATC and PSCCTh are presented in **Table 1**. There were no differences in age distribution, sex, ethnicity, marital status, or distribution of proximal metastasis between the two cancers. A higher percentage of patients with ATC underwent radiotherapy, chemotherapy, or surgery, and had multiple primary tumors and distant metastasis (p < 0.05).

**Table 1:** Analysis of differences in demographic and clinical characteristics between ATC and PSCCTh.

Characteristic	ATC (n = 1164) No. (%)	PSCCTh (n = 124) No. (%)	χ^2^	p
**Age (years)**			0.387	0.534
< 65	375 (32.2)	36 (29.0)		
≥ 65	789 (67.8)	88 (71.0)		
**Ethnicity**			1.261	0.532
Black	97 (8.3)	7 (5.6)		
Other	142 (12.2)	14 (11.3)		
White	925 (79.5)	103 (83.1)		
**Sex**			0.748	0.387
Female	718 (61.7)	71 (57.3)		
Male	446 (38.3)	53 (42.7)		
**Radiotherapy**			5.728	0.017
No/Unknown	530 (45.5)	71 (57.3)		
Yes	634 (54.5)	53 (42.7)		
**Chemotherapy**			4.876	0.027
No/Unknown	683 (58.7)	86 (69.4)		
Yes	481 (41.3)	38 (30.6)		
**Primary tumor**			4.792	0.029
One	276 (23.7)	41 (33.1)		
More than one	888 (72.3)	83 (66.9)		
**Marital status**			0.018	0.895
Married	632 (54.3)	66 (53.2)		
Other	532 (45.7)	58 (46.8)		
**Surgery**			7.771	0.005
No/Unknown	631 (54.2)	84 (67.7)		
Yes	533 (45.8)	40 (32.3)		
**N stage**			4.466	0.107
N0	425 (36.5)	48 (38.7)		
N1	566 (48.6)	50 (40.3)		
NX	173 (14.9)	26 (21.0)		
**M stage**			19.019	<0.001
M0	557 (47.9)	77 (62.1)		
M1	512 (44.0)	30 (24.2)		
MX	95 (8.1)	17 (13.7)		

ATC, Anaplastic thyroid carcinoma; PSCCTh, primary squamous cell carcinoma of the thyroid.

### Differences in CSS Between Patients With ATC and PSCCTh

**Figure 1:**
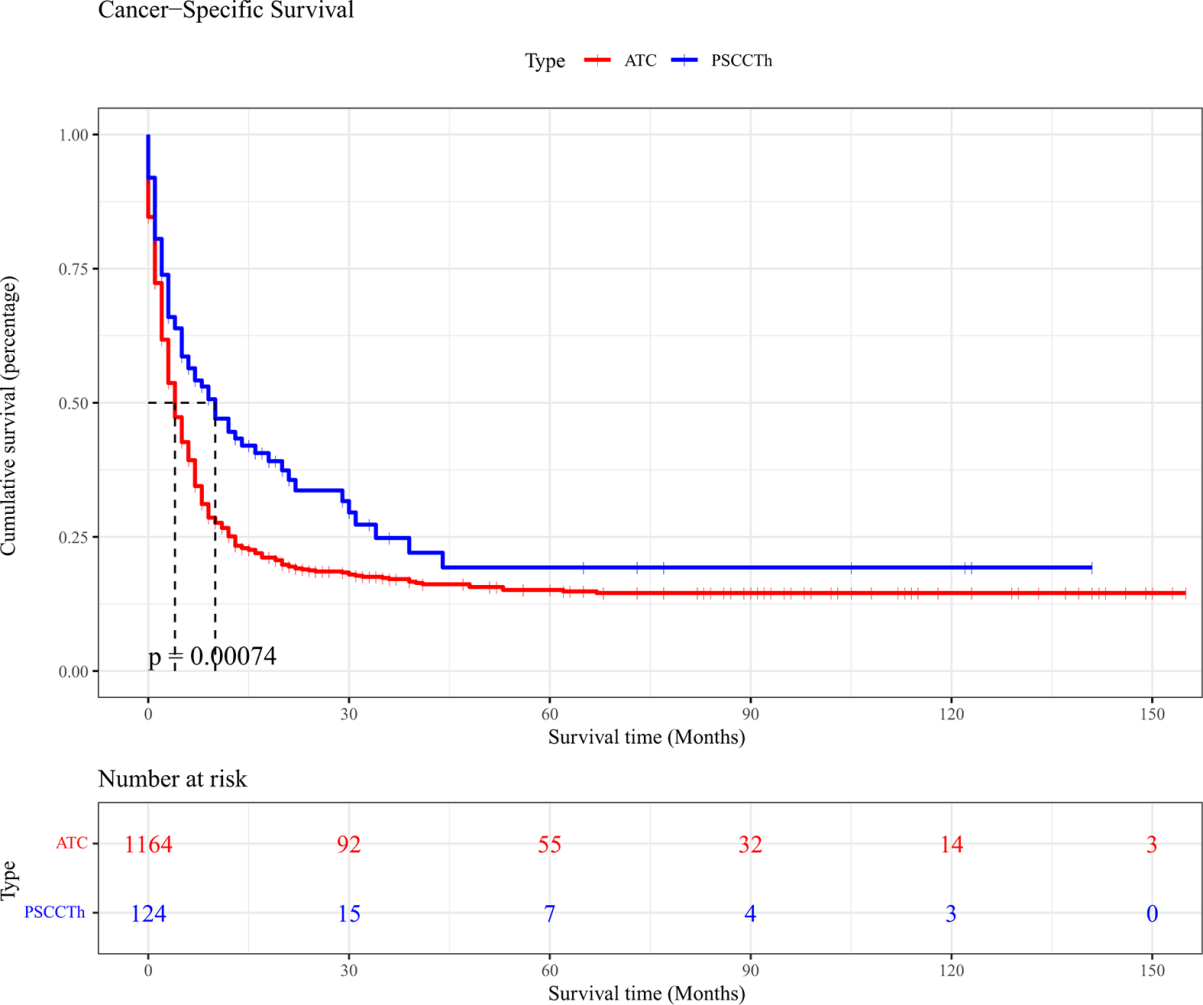
Kaplan-Meier curves of cancer-specific survival in patients with ATC and PSCCTh.

To evaluate the CSS prognosis of patients with ATC and PSCCTh, we visualized and analyzed CSS in patients from these two groups. Kaplan-Meier curves indicated that the median CSS of patients with ATC was 4 months, while that of patients with PSCCTh was almost 10 months. Log-rank test analysis demonstrated that CSS was longer in patients with PSCCTh than in those with ATC (p < 0.01) (**Figure 1**).

### Analysis of Prognostic Factors for CSS in Patients With ATC

Univariate Cox regression analysis of all variables was conducted to investigate the influence of different factors on CSS prognosis in patients with ATC, and showed that ‘white’ ethnicity, radiotherapy, chemotherapy, surgery, and marital status were prognostic factors influencing CSS in patients with ATC. In multivariate Cox analysis, age ≥ 65 years, male sex, radiotherapy, chemotherapy, surgery, multiple primary tumors, and distant metastasis were independent prognostic factors affecting CSS in patients with ATC (**Table 2**). The C-index of the model was 0.803 (95% confidence interval (CI): 0.787–0.819), demonstrating that it has a good discriminative ability.

**Table 2:** Univariate and multivariate Cox regression models of factors associated with CSS in patients with ATC.

Characteristic	Univariate			Multivariate		
	HR	95% CI	p	HR	95% CI	p
**Age (years)**						
< 65	Reference			Reference		
≥ 65	1.157	0.999–1.339	0.051	1.291	1.109–1.503	<0.001
**Ethnicity**						
Black	Reference					
Other	0.957	0.710–1.290	0.773			
White	0.765	0.601–0.973	0.029			
**Sex**						
Female	Reference			Reference		
Male	0.891	0.772–1.029	0.118	0.581	0.341–0.992	0.047
**Radiotherapy**						
No/Unknown	Reference			Reference		
Yes	0.460	0.399–0.531	<0.001	0.550	0.470–0.644	<0.001
**Chemotherapy**						
No/Unknown	Reference			Reference		
Yes	0.534	0.463–0.617	<0.001	0.655	0.557–0.771	<0.001
**Primary tumor**						
More than one	Reference			Reference		
One	12.950	8.476–19.790	<0.001	13.937	9.108–21.326	<0.001
**Marital status**						
Married	Reference			Reference		
Other	1.262	1.097–1.451	0.001	1.272	1.102–1.469	0.001
**Surgery**						
No/Unknown	Reference			Reference		
Yes	0.434	0.375–0.502	<0.001	0.516	0.443–0.600	<0.001
**N stage**						
N0	Reference					
N1	1.318	1.128–1.539	<0.001			
NX	1.580	1.275–1.958	<0.001			
**M stage**						
M0	Reference			Reference		
M1	2.428	2.089–2.823	<0.001	1.991	1.698–2.334	<0.001
MX	1.546	1.176–2.032	0.002	1.202	0.910–1.589	0.195

ATC, Anaplastic thyroid carcinoma.

### Analysis of Prognostic Factors for CSS in Patients With PSCCTh

To study influences on CSS in patients with PSCCTh, we conducted univariate and multivariate Cox model analyses of various potential prognostic factors. Univariate Cox analysis found that age, sex, and multiple primary tumors were factors influencing CSS in patients with PSCCTh. Although radiotherapy and chemotherapy were not significantly associated according to univariate tests, they are routine treatments for PSCCTh; therefore, we included them in the multivariate model. The multi-factor Cox model indicated that age, radiotherapy, multiple primary tumors, and surgery were independent prognostic factors for CSS in patients with PSCCTh. Distant metastasis did not retain significance in the multivariate model; however, its test value was close to the significance level (**Table 3**). The C-index of the model was 0.829 (95% CI: 0.786–0.872), indicating that it has good discrimination ability for patients with PSCCTh.

**Table 3:** Univariate and multivariate Cox regression model of factors associated with CSS in patients with PSCCTh.

Characteristic	Univariate			Multivariate		
	HR	95% CI	p	HR	95% CI	p
**Age (years)**						
< 65	Reference			Reference		
≥ 65	1.930	1.134–3.286	0.015	2.033	1.141–3.621	0.016
**Ethnicity**						
Black	Reference					
Other	1.021	0.313–3.324	0.973			
White	0.958	0.367–2.644	0.933			
**Sex**						
Female	Reference					
Male	0.546	0.329–0.905	0.019			
**Radiotherapy**						
No/Unknown	Reference			Reference		
Yes	0.962	0.607–1.569	0.921	0.529	0.314–0.889	0.016
**Chemotherapy**						
No/Unknown	Reference					
Yes	1.036	0.638–1.682	0.887			
**Primary tumor**						
More than one	Reference			Reference		
One	33.640	4.669–242.400	< 0.001	48.055	6.593–350.287	< 0.001
**Marital status**						
Married	Reference					
Other	1.427	0.890–2.286	0.140			
**Surgery**						
No/Unknown	Reference			Reference		
Yes	0.741	0.450–1.218	0.237	0.534	0.318–0.897	0.018
**N stage**						
N0	Reference					
N1	1.305	0.763–2.234	0.331			
NX	1.276	0.679–2.401	0.449			
**M stage**						
M0	Reference			Reference		
M1	1.342	0.796–2.265	0.270	1.544	0.883–2.697	0.127
MX	0.977	0.218–1.228	0.135	0.412	0.168–1.007	0.052

PSCCTh, primary squamous cell carcinoma of the thyroid.

## Discussion

Both ATC and PSCCTh are rare malignant thyroid tumors. The data analyzed in this study were all retrieved from the SEER database. The study period was from 2004 to 2015, and the study included 1164 and 124 patients with ATC and PSCCTh, respectively. Our results indicate that median CSS time was longer in patients with PSCCTh than in those with ATC, consistent with previous studies (8). We also found that a higher proportion of patients with ATC had distant metastases, consistent with the results reported by Glaser et al. (9).

In line with previous studies, we found that women comprise a higher proportion of patients with ATC and PSCCTh than men, which may be related to the biological properties of these cancers (10, 11). Since PSCCTh is a rare type of thyroid cancer histology and clinical experience of this disease is limited, appropriate treatment for PSCCTh is controversial. At present, the treatment methods applied for PSCCTh and ATC are similar, and primarily comprise chemotherapy, radiotherapy, and surgery; however, treatment rates for ATC were higher than those for PSCCTh, because compared with PSCCTh, ATC has a clear, well-defined structure (8).

Age was a prognostic factor for CSS in patients with ATC. The survival rate of patients ≥ 65 years old was less than that of patients < 65 years old, confirming the findings of Gui et al. (12). Smallridge et al. (13) demonstrated that, for patients diagnosed with early ATC, surgical treatment, chemotherapy, radiotherapy, and systemic treatment can achieve optimal survival outcomes, while active palliative and clinical care are crucial for individuals with advanced ATC (14). Relevant studies (15, 16) have reported that sex, surgical treatment, radiotherapy, and chemotherapy are all prognostic factors for CSS in patients with ATC, consistent with the results of this study. Here, we found that marital status was a prognosis factor for CSS in patients with ATC. Several studies have demonstrated that people with differing marital status have varying levels of home care and psychological support, resulting in differences in their living conditions (17, 18).

Our research demonstrates that PSCCTh is more likely to occur in elderly female patients. Age is an independent factor influencing CSS in patients with PSCCTh, whereas sex and ethnicity are not associated with prognosis. Radiotherapy is a protective factor for CSS in patients with PSCCTh, and related studies (19) have reached the same conclusion. In a case report, Shimaoka and Tsukada (20) proposed that chemotherapy has an active role in the treatment of PSCCTh; however, in this study we did not detect chemotherapy as a prognostic factor for CSS in patients with PSCCTh. Surgical treatment was also an independent factor which affected CSS in patients with PSCCTh. Published evidence indicates that the best treatment for PSCCTh is early diagnosis, followed by aggressive surgery to prevent bleeding or suffocation caused by obstruction (21). Unlike CSS of patients with ATC, marital status was not a prognostic factor for CSS in patients with PSCCTh. In the PSCCTh CSS prognostic model, the statistical test value of M stage is close to the threshold for statistical significance, and the hazard ratio (HR) value of Primary tumor is very large. In addition to the real role of the variables, the reason for this phenomenon may be caused by the sample size.

According to the American Thyroid Cancer Association guidelines, surgery is suitable for low-risk patients with small tumors (< 1 cm), without clinical lymph node metastases, because of its manageable risk (22). Conzo et al. found that patients who undergo hemithyroidectomy for thyroid follicular neoplasms had a lower incidence of hypothyroidism and shorter hospital stay than those receiving total thyroidectomy; however, total thyroidectomy should be the first choice in patients with high cancer risk (23). For patients with papillary thyroid carcinoma without lymph node involvement, prophylactic neck dissection is inappropriate, and there is controversy over whether radioactive iodine treatment is necessary (24). Pezzolla et al. suggested that incidental thyroid carcinomas with a predominant papillary pattern can be treated with endocrine surgery after clear follow-up by a multidisciplinary follow-up team (25). For ATC, Conzo et al. (26) proposed that surgery should be the basis of treatment, with multimodal treatment, including surgery, chemotherapy, and radiotherapy, having potential to improve survival of patients with locally advanced ATC, and our study also reached similar conclusions. Research by Au et al. (27) found that surgery is not a prognostic factor for CSS in patients with PSCCTh; this differs from our results, as our findings indicate that surgical treatment is protective factor for CSS in patients with PSCCTh, possibly due to variations in the surgical and radiological methods used.

Lin et al. (15) found that, although the overall survival of patients with multiple primary thyroid tumors was much lower than that of patients with a single primary thyroid cancer, there was no difference in CSS between patients with multiple and single primary thyroid tumors. Amer (28) compared the survival rates of patients with single and multiple primary tumors, and found that the overall survival rate of patients with multiple primary tumors was higher than that of patients with single primary tumors. This may be because the survival time of patients with many types of cancer is not sufficiently long to develop a second origin. In this study, multiple primary tumors was a protective factor for CSS in patients with ATC and PSCCTh; this does not indicate that the mortality rate of patients with single primary thyroid tumors is lower and may reflect that the mortality rate from other cancers in patients with several primary tumors is higher than that caused by thyroid cancer.

This study has some shortcomings. The limited number of patients with PSCCTh included in the study (N = 124) may have contributed to possible false negative results. In particular, some factors had large HR values in the model, and comprehensive validation methods could not be applied, with only C-index calculation used. In addition, this was a retrospective study and some samples with incomplete data were removed, with only patients with complete information included, which may have led to bias.

## Conclusion

This study included analysis of CSS in a large population cohort of patients with ATC and PSCCTh. Our findings show that, although both ATC and PSCCTh are aggressive thyroid malignancies, the prognosis for CSS is better in patients with PSCCTh than in those with ATC. We have established prognostic models for CSS of patients with both ATC and PSCCTh, and the prognostic factors for the two diseases were not identical, indicating that specific clinical treatment and management plans need to be developed for these two types of thyroid cancer.

## Data Availability Statement

The raw data supporting the conclusions of this article will be made available by the authors, without undue reservation.

## Ethics Statement

Ethical review and approval was not required for the study on human participants in accordance with the local legislation and institutional requirements. Written informed consent for participation was not required for this study in accordance with the national legislation and the institutional requirements.

## Author Contributions

SJ and Y-NY contributed to the conception and design of the study. SJ and XML collected and analyzed data. DHL and DDP wrote the manuscript. All authors contributed to the article and approved the submitted version.

## Funding

This study was supported by funds from the National Natural Science Foundation of China (61803112, 32160151), the Ministry of Education Industry-Academia Cooperation Collaborative Education Project (202101311012, 202102576027), the Science and Technology Foundation of Guizhou Province (2019-2811, 2018-1133), NSFC Incubation Program by Guizhou Medical University (20NSP033).

## Conflict of Interest

The authors declare that the research was conducted in the absence of any commercial or financial relationships that could be construed as a potential conflict of interest.

## Publisher’s Note

All claims expressed in this article are solely those of the authors and do not necessarily represent those of their affiliated organizations, or those of the publisher, the editors and the reviewers. Any product that may be evaluated in this article, or claim that may be made by its manufacturer, is not guaranteed or endorsed by the publisher.
